# Self-efficacy of emergency management of domestic helpers in pediatric home accidents: A cross-sectional survey in Hong Kong

**DOI:** 10.3389/fped.2022.997834

**Published:** 2022-10-19

**Authors:** Jonathan Ka-Ming Ho, Jessie Yuk-Seng Chung, Shu-Nam Cheung, Winnie Wing-Yan Pang, Po-Yi Yau, Simon Ching Lam

**Affiliations:** ^1^ School of Nursing and Health Studies, Hong Kong Metropolitan University, Hong Kong SAR, China; ^2^ School of Nursing, Tung Wah College, Hong Kong SAR, China; ^3^ Surgical Unit, Ruttonjee Hospital, Hong Kong SAR, China; ^4^ In-Patient Unit, Bradbury Hospice, Hong Kong SAR, China; ^5^ Medical Unit, Tseung Kwan O Hospital, Hong Kong SAR, China

**Keywords:** self-efficacy, emergencies, first aid, child, home accidents, caregivers

## Abstract

**Background:**

Accidental injuries are the leading cause of deaths and disabilities in children globally and most of them occur at home. To save life and prevent sequelae, domestic helpers (DHs) require providing emergency management (i.e., first aid) to children involved in home accidents. However, their self-efficacy in emergency management for children is rarely investigated. Hence, this study aimed to tap that research gap.

**Methods:**

This study adopted a cross-sectional descriptive survey design. A convenience sample of 385 DHs was obtained in Hong Kong. DHs' self-efficacy in emergency management for children involved in home accidents was measured using a 12-item well-validated survey instrument “Self-Efficacy of First Aid in Unintentional Injury at Home”. The total score ranged from 0 to 48. A higher score indicates greater confidence in emergency management for children involved in home accidents.

**Results:**

All the participants were women and most of them were aged between 31 and 35 years (*N* = 103, 26.8%). The mean score for DHs' self-efficacy in emergency management was 29.0 (SD 10.1). The three items with the lowest self-efficacy were managing bone fractures, performing cardiopulmonary resuscitation, and providing artificial respiration. Bivariate analysis showed that DHs' self-efficacy was significantly related to their educational level, first aid training, caring experience, and working experience. Multiple linear regression indicated that DHs' educational level (*β* = 0.136, *p* = 0.001) and first aid training (*β *= 0.532, *p* < 0.001) were significantly predicting their self-efficacy.

**Conclusion:**

DH's self-efficacy of emergency management for children involved in home accidents was low, particularly in those severe situations and complicated first aid procedures.

## Introduction

According to the World Health Organization, accidental injuries are the leading cause of deaths and disabilities in children globally. More than 2,000 children die from accidental injuries every day, and tens of millions of children are hospitalised with potential disabilities every year (1, 2). Accidental injuries impose a burden on the healthcare system and may severely affect the family income and children's quality of life (2). The majority of accidental injuries occur at home where children spend the most of their time (3). Emergency management must be provided to children with accidental injuries before further treatment is available (4).

First aid is defined as the emergency management provided to an injured or ill person using readily available resources. The goals of emergency management are to preserve life, prevent deterioration, and promote recovery for the casualty (5). Providing immediate and appropriate emergency management to children with accidental injuries may reduce the risk of death by 25% to 35% (6). First aid providers are usually nearby individuals without medical training. To save life and prevent sequelae, parents and caregivers who are the closest to children should be capable of providing emergency management to children involved in home accidents (7).

Self-efficacy refers to individuals' confidence in their capability to engage in certain behaviours to achieve specific levels of performance. Individuals with high self-efficacy view difficult tasks as challenges to be overcome, whereas those with low self-efficacy view difficult tasks as threats to be avoided (8). Evaluating self-efficacy can help predict related behaviours when measuring actual behaviours is not feasible. Limited evidence is available regarding the self-efficacy of providing emergency management to children involved in home accidents although it can reflect the performance of providing emergency management to children with accidental injuries. A study reported that parents had insufficient self-efficacy to provide emergency management to children involved in home accidents and lacked the confidence to perform cardiopulmonary resuscitation (CPR) (7). However, no similar study has been conducted on domestic helpers (DHs) who are common caregivers for many children in several countries, such as North America, Arab States, Europe, Asia, and the Pacific. In 2015, more than 67 million DHs, 11.5 million of whom were migrant DHs, were employed globally to perform household duties, care for children, and conduct other tasks (9, 10). The acquisition of a first aid certificate is not compulsory for the employment of DHs. The acquirement of first aid knowledge among DHs who did not have a certificate depends on the subsidy of such training by their employers. It was found that only one-third of DHs had received first aid training and thus had poor knowledge related to emergency management (11). This study investigated DHs' self-efficacy of providing emergency management to children involved in home accidents.

## Methods

### Design

This study adopted a cross-sectional descriptive survey design. The Strengthening the Reporting of Observational Studies in Epidemiology (STROBE) checklist was used to identify all essential items in this study (Supplementary Appendix 1).

### Sample

A convenience sample of 385 DHs was recruited from Central, Causeway Bay, Tsuen Wan, and Mong Kok districts where DHs usually gathered in Hong Kong. DHs who worked in a family with at least one child (aged <18 years) were eligible for inclusion in this study. DHs who could not read and communicate in English were excluded from this study.

The required sample size was estimated using a statistical formula $\left(\mathrm{sample}\;\mathrm{size}=Z_{1-\alpha /2}^{2}p(1-p)/d^{2}\right)$ based on the population size of DHs, margin of error, and confidence interval (CI) (12). The population size of DHs was approximately 390,000 in Hong Kong (13). $Z_{1-\alpha /2}$ was the standard normal variate and was equal to 1.96 at 95% CI. *p* was the expected proportion of DHs having a medium level of self-efficacy and was assumed as 0.5 to generate the largest sample size. *d* was the margin of error and was set at 5% that was acceptable in most studies (12). Therefore, the sample size should be at least 384, which was obtained through the insertion of the aforementioned values into the formula.

### Measurement

Data were collected using a questionnaire composed of an information sheet, a demographic data sheet, and a survey instrument “Self-Efficacy of First Aid in Unintentional Injury at Home” adopted from the study of Wei et al. (7). Wei and colleagues developed a 37-item English instrument from a synthesized literature review of emergency management and divided it into a 25-item subscale consisting of the knowledge of emergency management and a 12-item subscale focusing on the self-efficacy regarding emergency management. Thereafter the 37-item English instrument was translated into a Chinese version. This study utilized the 12-item English subscale because the majority of DHs can speak and comprehend English and the subscale measures the self-efficacy of providing emergency management to children involved in home accidents that covers the management of scalds, choking, respiratory arrest, cardiac arrest, bone fractures, abrasions, nosebleeds, muscle strain, drowning, and poisoning. The participants were asked to rate each item on a 5-point Likert scale, with 4 indicating “very sure” (100% certainty) and 0 indicating “not sure” (0% certainty). Thus, the total score ranged from 0 to 48. A higher score indicates greater confidence in providing emergency management to children involved in home accidents (7). The content validity and internal consistency of the 37-item Chinese instrument were examined in the study of Wei et al. (7). The content validity index was 0.97, which indicated high content validity (7, 14). The Cronbach's alpha values of the 25-item Chinese subscale were 0.70 and 0.69 in the pilot study (*N* = 34) and main study (*N* = 445) of Wei et al. (7), respectively, which indicated acceptable internal consistency (14). Additionally, the Cronbach's alpha values of the 12-item Chinese subscale were 0.91 and 0.89 in the pilot study (*N* = 34) and main study (*N* = 445) of Wei et al. (7), respectively, which indicated high internal consistency (14). This study ascertained the content validity and internal consistency of the 12-item English subscale. A content validity index of 0.99, which indicated high content validity, was determined by an expert panel comprising two physicians, two emergency nurses, and two auxiliary medical service providers. Cronbach's alpha values of 0.89 and 0.86, which indicated high internal consistency, were demonstrated in the pilot study (*N* = 53) and main study (*N* = 385), respectively (14).

### Data collection

First, the investigators identified and approached the DHs who usually gathered into groups outside the buildings, under the footbridges, and inside the parks in Central, Causeway Bay, Tsuen Wan, and Mong Kok districts. Second, the investigators introduced themselves and explained the background and aim of this study to the DHs by using an information sheet. Third, the DHs who were eligible according to the inclusion and exclusion criteria were invited to participate in this study. Fourth, the DHs self-administered the questionnaire and received assistance from the investigators, if required. This study was approved by the Research Ethics Committee of The Open University of Hong Kong. Informed consent was obtained for all participants before the data collection.

### Data analysis

Data were analysed using the Statistical Package for the Social Sciences (SPSS) for Windows (version 22.0). Descriptive statistics, including the frequency, percentage, mean, and standard deviation (SD), were used to describe the participants’ personal characteristics and their self-efficacy of providing emergency management to children involved in home accidents. Bivariate analysis, namely the independent samples *t* test, one-way analysis of variance, and Spearman's Rho, were used to examine the relationships between the participants' personal characteristics and their self-efficacy of providing emergency management to children involved in home accidents. Regression analysis, namely the multiple linear regression, was used to examine the predictors for the participants' self-efficacy of providing emergency management to children involved in home accidents. A *p*-value of <0.05 was considered statistically significant.

## Results

### Personal characteristics of the participants

Table 1 lists the personal characteristics of the participants. All the participants were women (*N* = 385, 100%). The majority of the participants were aged between 31 and 35 years (*N* = 103, 26.8%), and only 19 (4.9%) participants were aged >50 years. Nearly all the participants had a tertiary (*N* = 190, 49.4%) or secondary (*N* = 181, 47.0%) educational level, with the remaining having a primary educational level (*N* = 10, 2.6%) or below (*N* = 4, 1.0%). The majority of the participants were married (*N* = 226, 58.7%), and the remaining were single (*N* = 143, 37.1%), divorced (*N* = 4, 1.0%), or widowed (*N* = 12, 3.1%). Around half of the participants had completed first aid training (*N* = 207, 53.8%), and less than half of the participants had previous experience of managing children involved in home accidents (*N* = 161, 41.8%). The mean number of years caring for children was 7.1 (SD 5.8) years, and the mean number of years working as a DH was 7.2 (SD 6.3) years.

**Table 1:** Personal characteristics of the participants (*N* = 385).

	*N* (%)
**Gender**	
Male	0 (0)
Female	385 (100)
**Age group (years)**	
25–30	81 (21.0)
31–35	103 (26.8)
36–40	85 (22.1)
41–45	68 (17.7)
46–50	29 (7.5)
Above 50	19 (4.9)
**Educational level**	
Below primary	4 (1.0)
Primary	10 (2.6)
Secondary	181 (47.0)
Tertiary	190 (49.4)
**Marital status**	
Married	226 (58.7)
Single	143 (37.1)
Divorced	4 (1.0)
Widowed	12 (3.1)
**Completeness of first aid training**	
Yes	207 (53.8)
No	178 (46.2)
**Previous experience of child home accidents**	
Yes	161 (41.8)
No	224 (58.2)
Experience of caring for children (years)	7.1 (5.8)^a^
Working experience as a domestic helper (years)	7.2 (6.3)^a^

*N*, number.

^a^ Data are presented in mean (standard deviation).

### Self-efficacy of providing emergency management to children involved in home accidents

Table 2 presents the participants' self-efficacy of providing emergency management to children involved in home accidents. The mean score for the self-efficacy of providing emergency management was 29.0 (SD 10.1). Approximately one-third of the participants scored less than 24 points (*N* = 120, 31.2%), which was half of the total score. Only 7 participants (1.8%) rated “100% certainty” in all the items. The three items with the highest mean score for the self-efficacy were (1) “I know how to call 999 for help during an accident” (mean score: 3.67, SD 0.72), (2) “I can perform “flush, remove, soak, cover, and send” when a child is scalded” (mean score: 2.87, SD 1.06), and (3) “I can stop bleeding when a child has a nosebleed” (mean score: 2.84, SD 1.14). The three items with the lowest mean score for the self-efficacy were (1) “I can fix the injured area when a child has a bone fracture” (mean score: 1.32, SD 1.27), (2) “I can perform CPR when a child has no heartbeat” (mean score: 1.77, SD 1.37), and (3) “I can perform artificial respiration when a child is not breathing” (mean score 2.16, SD 1.25).

**Table 2:** Self-efficacy of providing emergency management to children involved in home accidents (*N* = 385).

	*N* (%)					Mean (SD)	Rank
	100% Certainty	70% Certainty	50% Certainty	30% Certainty	0% Certainty		
1. I know how to call 999^a^ for help during an accident.	301 (78.2)	55 (14.3)	20 (5.2)	6 (1.6)	3 (0.8)	3.67 (0.72)	1
2. I can perform “flush, remove, soak, cover, and send” when a child is scalded.	127 (33.0)	140 (36.4)	70 (18.2)	38 (9.9)	10 (2.6)	2.87 (1.06)	2
3. I can perform first aid (Heimlich manoeuvre) when a child is choking.	114 (29.6)	136 (35.3)	78 (20.3)	39 (10.1)	18 (4.7)	2.75 (1.13)	4
4. I can perform artificial respiration when a child is not breathing.	58 (15.1)	110 (28.6)	104 (27.0)	61 (15.8)	52 (13.5)	2.16 (1.25)	10
5. I can perform cardiopulmonary resuscitation when a child has no heartbeat.	51 (13.2)	70 (18.2)	104 (27.0)	58 (15.1)	102 (26.5)	1.77 (1.37)	11
6. I can fix the injured area when a child has a bone fracture.	24 (6.2)	53 (13.8)	91 (23.60	72 (18.7)	145 (37.7)	1.32 (1.27)	12
7. I can address wounds when a child has abrasions.	98 (25.5)	95 (24.7)	98 (25.5)	59 (15.3)	35 (9.1)	2.42 (1.27)	6
8. I can stop bleeding when a child has a nosebleed.	142 (36.9)	105 (27.3)	89 (23.1)	33 (8.6)	16 (4.2)	2.84 (1.14)	3
9. I can address the injured area when a child has a muscle strain.	61 (15.8)	115 (29.9)	100 (26.0)	60 (15.6)	49 (12.7)	2.20 (1.25)	7
10. I can perform first aid when a young child is drowning.	61 (15.8)	109 (28.3)	106 (27.5)	58 (15.1)	51 (13.2)	2.18 (1.25)	8
11. I can perform correct methods when a child eats something by mistake.	105 (27.3)	122 (31.7)	94 (24.4)	40 (10.4)	24 (6.2)	2.63 (1.17)	5
12. I can judge a child's injury status when an accident occurs.	64 (16.6)	92 (23.9)	115 (29.9)	68 (17.7)	46 (11.9)	2.17 (1.24)	9

*N*, number; SD, standard deviation.

^a^ 999 is the emergency phone number in The Government of Hong Kong Special Administrative Region.

### Bivariate analysis of the relationships between personal characteristics and self-efficacy of providing emergency management to children involved in home accidents

Table 3 presents the bivariate analysis of the relationships between the participants’ personal characteristics and their self-efficacy of providing emergency management to children involved in home accidents. Since only a few participants had a primary educational level or below, the variables “below primary educational level”, “primary educational level”, and “secondary educational level” were re-categorized into a variable “secondary educational level or below” for the bivariate analysis. Similarly, the variables “widowed”, “divorced”, and “single” were re-categorized into a variable “single at present” for the bivariate analysis because only a few participants were widowed or divorced. The mean score of the participants with a tertiary educational level (31.1, SD 9.2) was significantly higher than that of those with a secondary educational level or below (26.9, SD 10.6; *p* < 0.001). Moreover, the participants who completed first aid training (34.3, SD 7.4) had a significantly higher mean score than those who did not complete the training (22.8, SD 9.4; *p* < 0.001). In addition, the mean score of the participants who had previous experience of managing children involved in home accidents (31.2, SD 8.8) was significantly higher than that of those who did not have such experience (27.4, SD 10.8; *p* < 0.001). Furthermore, the mean number of years of experience caring for children (*r_s_* = 0.128, *p* = 0.012) and the mean number of years of experience working as a DH (*r_s_* = 0.116, *p* = 0.023) were significantly positively correlated with the mean score for the self-efficacy of providing emergency management. The remaining participants' personal characteristics were not significantly related to the mean score for the self-efficacy of providing emergency management.

**Table 3:** Bivariate analysis of the relationships between personal characteristics and self-efficacy of providing emergency management to children involved in home accidents (*N* = 385).

	Mean (SD)	*p*-value
**Age group (years)**		
25–30	28.5 (9.1)	0.694
31–35	27.9 (9.4)	
36–40	30.2 (10.5)	
41–45	28.9 (11.4)	
46–50	29.6 (11.1)	
Above 50	30.5 (10.8)	
**Educational level**		
Secondary or below	26.9 (10.6)	<0.001*
Tertiary	31.1 (9.2)	
**Marital status**		
Married	28.6 (10.4)	0.361
Single at present	29.6 (9.7)	
**Completeness of first aid training**		
Yes	34.3 (7.4)	0.001*
No	22.8 (9.4)	
**Previous experience of child home accidents**		
Yes	31.2 (8.8)	0.001*
No	27.4 (10.8)	
Experience of caring for children (years)	*r*_s_ = 0.128^a^	0.012*
Working experience as a domestic helper (years)	*r*_s_ = 0.116^a^	0.023*

SD, standard deviation.

^a^ Relationship is presented in Spearman's Rho cofficient.

* Statistically significant (*p* < 0.05).

### Multiple linear regression of the predictors for self-efficacy of providing emergency management to children involved in home accidents

Table 4 presents the multiple linear regression of the predictors for the participants' self-efficacy of providing emergency management to children involved in home accidents. The participants' personal characteristics (educational level, completeness of first aid training, previous experience of child home accidents, experience of caring for children, and working experience as a DH), which had a significant relationship with their mean score for the self-efficacy of providing emergency management and met the assumptions of multiple linear regression, were entered into the model. The adjusted *R*^2^ was 0.344. It was estimated that a one-unit increase in the educational level was associated with a 0.136-unit increase in the mean score for the self-efficacy of providing emergency management (*β *= 0.136, *p* = 0.001). Moreover, a one-unit increase in the completeness of first aid training was associated with a 0.532-unit increase in the mean score for the self-efficacy of providing emergency management (*β *= 0.532, *p* < 0.001).

**Table 4:** Multiple linear regression of the predictors for self-efficacy of providing emergency management to children involved in home accidents (*N* = 385).

	*B*	SE	*β*	95% CI	*p*-value
(Constant)	17.378	1.501	–	14.427–20.329	<0.001*
Educational level	2.744	0.855	0.136	1.064–4.425	0.001*
Completeness of first aid training	10.796	0.861	0.532	9.102–12.489	<0.001*
Previous experience of child home accidents	1.320	0.880	0.064	−0.409 to 3.050	0.134
Experience of caring for children	0.067	0.083	0.038	−0.096 to 0.231	0.417
Working experience as a domestic helper	0.094	0.075	0.058	−0.053 to 0.241	0.210

*β*, standardized coefficient; *B*, unstandardized coefficient; CI, confidence interval; SE, standard error.

* Statistically significant (*p* < 0.05).

## Discussion

To the best of our knowledge, this is the first study to investigate DHs' self-efficacy of providing emergency management to children involved in home accidents. Many parents have to work nowadays. Because of long working hours, a considerable number of children spend the major part of their day at home along with DHs. Thus, DHs play an essential role in caring for children. Accidents are the primary cause of injuries or even deaths in children (15). However, this study indicated that the DHs' self-efficacy of providing emergency management to children involved in home accidents was low; the mean score was 29.0 (SD 10.1) (maximum score = 48 points), with approximately one-third of the participants achieving less than half of the total score. Concordant with the present study's finding, Wei et al. (7) found that the parents' self-efficacy in emergency management for children involved in home accidents was low; the mean score was 30.3 (SD 9.2). These findings may have been due to the lack of first aid training among different kinds of caregivers. Only 35.3% of the parents and 53.8% of the DHs had received first aid training before participating in the studies. According to Bandura's social cognitive theory, an individual's self-efficacy is positively associated with performance (16). Therefore, DHs with lower self-efficacy of providing emergency management tend to be more frightened during emergencies, resulting in poorer performance in emergency management.

The three items with the highest self-efficacy of providing emergency management to children involved in home accidents were calling the emergency number, handling scalds, and stopping nosebleeds with the mean scores of 3.67 (SD 0.72), 2.87 (SD 1.06), and 2.84 (SD 1.14), respectively. Activating the emergency service, treating the scalded area, and controlling a nosebleed are relatively straightforward, requiring fewer specific techniques for DHs.

The DHs had the lowest self-efficacy for managing bone fractures, performing CPR, and providing artificial respiration to children involved in home accidents. The mean scores of these three items were only 1.32 (SD 1.27), 1.77 (SD 1.37), and 2.16 (SD 1.25), respectively. The low scores would be due to the requirement of more specific techniques for handling these urgent situations. For instance, a first aider should know how to immobilise the fractured part and stop any bleeding before sending the victim to the hospital, locate the optimal chest compression point for CPR and compress fast and deep enough to maintain blood flow throughout the body, and provide positive pressure breaths to produce a visible chest rise (17).

Bone fractures are among the most common injuries in children. Approximately 30% of children experience at least one fracture throughout their childhood (18). Upper extremity fractures are frequent fall-related injuries in young children. Because of immature developments in their motor abilities, they tend to fall off furniture and playground equipment (19). In addition, femur fractures are the most common type of fractures requiring hospitalisation in children (20). Therefore, the correct emergency management of fractures is essential because it can reduce pain and prevent further damage in children with these injuries. Sudden cardiac arrests are rare but often fatal in children, and the majority of cases occur due to asphyxia (21). Children with pneumonia, trauma, or aspiration may develop acute respiratory distress syndrome, which results in breathing failure and decreased oxygen levels (22). Although administering CPR and providing artificial respiration are less required in children, these procedures may save a child's life when the heart stops beating or the lungs stop working. However, the DHs' self-efficacy in the aforementioned aspects was considerably poor; thus, comprehensive first aid training should be provided to DHs.

This study demonstrated that educational level and completeness of first aid training are predictors for the DHs' self-efficacy of providing emergency management to children involved in home accidents. The participants with a tertiary educational level had higher self-efficacy of providing emergency management. Contrary to the finding of this study, Wei et al. (7) observed that no significant relationship existed between educational level and parents' self-efficacy of providing emergency management. Besides, The participants who completed first aid training had higher self-efficacy of providing emergency management. Consistent with the present study's finding, Wei et al. (7) suggested that first aid training and knowledge were significantly related to parents' self-efficacy of providing emergency management. First aid training improves one's knowledge and promotes the use of proper techniques to handle injuries (23). Therefore, first aid training empowers individuals and increases their propensity to implement protective measures for wounded individuals. Nearly half of the participants in this study did not attend any formal first aid training. This might explain their low self-efficacy of providing emergency management to children involved in home accidents.

This study showed that previous experience of child home accidents, experience of caring for children, and working experience as a DH are not predictors for the DHs' self-efficacy of providing emergency management to children involved in home accidents. We should know that having working experience as a DH doesn't mean having experience of caring for children; having experience of caring for children doesn't mean having previous experience of child home accidents; having previous experience of child home accidents doesn't mean acquiring appropriate and sufficient knowledge and skills in emergency management. Conversely, unpleasant experiences, such as failure to fix a bone fracture, may lower the DHs' confidence in emergency management for children involved in home accidents.

### Strengths and limitations

This study has some strengths and limitations. We included a sufficiently large sample from four districts in Hong Kong to ensure the statistical power of this study. However, DHs who could not read and communicate in English were excluded so that the findings of this study may not be generalized to them. Besides, the use of self-reported data may lead to social desirability response bias because the participants tended to answer questions in a manner that would be considered preferable by the public (14). Thus, future studies should perform knowledge and skill assessments to obtain more objective and comprehensive information on DHs’ performance in providing emergency management to children involved in home accidents.

### Implications

This study has some practical implications. Every DH should undergo training in the emergency management of children involved in home accidents. Studies have encouraged parents and school teachers to learn emergency management (4, 5); however, limited advice is provided for DHs who play a significant role in childcare. Therefore, first aid training focusing on bone fracture management and basic life support is required, particularly for DHs who care for children. Li et al. (24) reported that the interactive training strategy was the most effective for first aid training. They suggested that lectures with interactive discussion, small group activity, and scenario-based learning facilitate learners' knowledge retention. Moreover, scenario-based training creates more opportunities for learners to practice first aid techniques to handle different situations, thus improving their self-efficacy and ability in emergency management (25).

Recommendations for future research include examining DHs' actual knowledge and skills to obtain more comprehensive information on their ability of providing emergency management to children involved in home accidents and design the first-aid training content. For example, in this study, 36.9% of the participants rated 100% certainty in stopping bleeding when a child has a nosebleed. However, whether they could demonstrate the correct technique to stop bleeding was not assessed. Al-Kubaisy et al. (26) reported that even if the participants claimed that they had adequate knowledge for stopping a nosebleed, they still ignored the importance of the site and duration of nasal compression. Further implementation research is required to determine the most effective methods for improving the self-efficacy and competence of DHs in managing children involved in home accidents.

## Conclusion

The DHs' self-efficacy of providing emergency management to children involved in home accidents was low. A significant relationship was observed between the self-efficacy of providing emergency management and completeness of first aid training. Therefore, first aid training emphasising bone fracture management and basic life support should be encouraged for DHs responsible for caring for children. Improving DHs' self-efficacy of providing emergency management to children involved in home accidents is crucial to prevent the deterioration of injuries and increase the likelihood of survival in children.

## Data Availability

The raw data supporting the conclusions of this article will be made available by the authors, without undue reservation.
